# Studies on Osteocytes in Their 3D Native Matrix Versus 2D In Vitro Models

**DOI:** 10.1007/s11914-019-00521-1

**Published:** 2019-06-25

**Authors:** Chen Zhang, Astrid D. Bakker, Jenneke Klein-Nulend, Nathalie Bravenboer

**Affiliations:** 1 Department of Oral Cell Biology, Amsterdam Movement Sciences, Academic Centre for Dentistry Amsterdam (ACTA), University of Amsterdam and Vrije Universiteit Amsterdam, Amsterdam, The Netherlands; 2 Department of Clinical Chemistry, Amsterdam Movement Sciences, Amsterdam UMC, Vrije Universiteit Amsterdam, Amsterdam, The Netherlands; 3 Department of Internal Medicine, Division of Endocrinology and Center for Bone Quality, Leiden University Medical Center, Leiden, The Netherlands

**Keywords:** Models, Native matrix, Niche, Osteocyte, Three-dimensional, Two-dimensional

## Abstract

**Purpose of Review:**

Osteocytes are responsible for mechanosensing and mechanotransduction in bone and play a crucial role in bone homeostasis. They are embedded in a calcified collagenous matrix and connected with each other through the lacuno-canalicular network. Due to this specific native environment, it is a challenge to isolate primary osteocytes without losing their specific characteristics in vitro. This review summarizes the commonly used and recently established models to study the function of osteocytes in vitro.

**Recent Findings:**

Osteocytes are mostly studied in monolayer culture, but recently, 3D models of osteocyte-like cells and primary osteocytes in vitro have been established as well. These models mimic the native environment of osteocytes and show superior osteocyte morphology and behavior, enabling the development of human disease models.

**Summary:**

Osteocyte-like cell lines as well as primary osteocytes isolated from bone are widely used to study the role of osteocytes in bone homeostasis. Both cells lines and primary cells are cultured in 2D-monolayer and 3D-models. The use of these models and their advantages and shortcomings are discussed in this review.

## Introduction

Osteocytes comprise 90–95% of all bone cells [1]. They are terminally differentiated cells derived from osteoblasts. There are about ten times more osteocytes than osteoblasts in bone [2], but the function and properties of osteocytes are still not fully unraveled. Mature osteocytes are embedded in hard mineralized matrix with their cell body residing in lacunae, and their dendritic cell processes running through canaliculi. The matrix immediately around the osteocyte cell body and processes is not calcified, and thus a three-dimensional (3D) network of lacunae and canaliculi exists containing non-mineralized, osteoid-like matrix and the osteocyte cells. Although osteocytes are embedded in calcified matrix, they communicate with other osteocytes, osteoblasts, osteoclasts, and blood vessels through their processes running through the canaliculi of the lacuno-canalicular network. Nutrient and oxygen transport occurs by interstitial fluid through this lacuno-canalicular system [3]. The importance of osteocyte has been underestimated until Skerry and colleagues reported in 1989 that osteocyte metabolism is affected by intermittent loading of bone [4]. Since then, osteocytes have been shown to play a major role in bone mechanosensation and mechanotransduction [5–8]. These processes are crucial for bone adaption to mechanical loading. During a lifetime, bone is continuously remodeled, which is regulated by mechanical loading. When bone is loaded, deformation of bone matrix induces a pressure gradient in the interstitial fluid surrounding the osteocytes, thereby generating fluid shear stress [9, 10]. The osteocyte processes are capable to sense this fluid shear stress [11]. Osteocytes then translate the mechanical stress into production of biochemical signaling molecules such as nitric oxide (NO) and prostaglandin E_2_ (PGE_2_) [8, 12, 13]. These signaling molecules regulate cellular activities in bone by modulating bone formation and bone resorption [14]. Expression of the osteocyte-specific protein sclerostin, which inhibits bone formation through blocking the Wnt signaling pathway, is suppressed by mechanical loading and induced during unloading [15••]. Osteocytes express osteoprotegerin (OPG) and NO in response to mechanical loading to suppress bone resorption, and macrophage-colony stimulating factor (M-CSF) and RANKL to promote osteoclast formation and bone loss under unloading [11, 15••, 16, 17].

Osteocytes also play an important role in regulating phosphate homeostasis, potentially functioning as endocrine cells [6, 17–19]. FGF23 is one of the most important osteocyte-secreted endocrine factors acting on the kidney by regulating phosphate levels in plasma through decreasing the reabsorption of phosphate and downregulation of 1α-hydroxylase expression, which is required for the production of the active vitamin D metabolite, 1,25-dihydroxyvitamin D (1,25(OH)_2_D) [6, 20, 21]. FGF23 is further known to decrease parathyroid hormone (PTH) secretion by acting on the parathyroid gland, while at the same time, PTH secretion increases FGF23 expression in vivo [20]. Osteocytes also produce other proteins that regulate phosphate homeostasis, such as dentin matrix protein 1 (DMP1), phosphate-regulating gene with homologies to endopeptidases on the X chromosome (PHEX), and matrix extracellular phosphoglycoprotein (MEPE) [22].

Osteocytes are important but mysterious bone cells, and therefore a solid in vitro osteocyte model is necessary to allow the investigation of the osteocyte function, i.e., their mechanoresponsiveness and endocrine function. Over the past two decades, several studies have been conducted on osteocytes in vitro, but these studies have some limitations. First, osteocytes are terminally differentiated, non-proliferative cells in vivo*.* For in vitro studies on osteocytes, cell proliferation is needed to obtain a sufficient cell number, but their proliferative capacity is generally low. Second, osteocytes in vivo are embedded in heavily mineralized extracellular matrix which affects both mechanosensation and mechanotransduction [1]. Third, the calcified matrix as micro-environment causes impeded O_2_ diffusion, which differs from regular cell culture conditions [23].

Currently, numerous attempts are made to improve culture models to study osteocytes. Several osteocyte cell lines have been established, and primary mouse and human osteocyte isolation protocols as well as culture models to simulate the native environment of osteocytes have been developed. The current review aims to provide an overview of these culture models to study osteocytes, with special focus on models used to determine the osteocyte mechano-response. We postulated that osteocytes cultured in their native matrix are superior to simulate natural osteocyte behavior and show a higher response to mechanical loading and broader expression of mechanically induced genes, compared to isolated osteocytes in monolayer cultures.

## Methods

We thoroughly searched the literature on 2D monolayer and 3D models to study osteocytes with the aim to provide an overview of these culture models to study osteocytes, with special focus on models used to determine the osteocyte mechano-response. The study methodology conformed to the Preferred Reporting Items for Systematic Reviews and Meta-Analyses Statement (PRISMA) for systematic reviews.

### Information Sources and Search

All articles were searched in the database PubMed and selected based on preset inclusion and exclusion criteria. Inclusion criteria were articles describing osteocyte culture methods using different culturing conditions, different cell types, and different interventions, using the following key words: osteocyte OR osteocyte cell line OR MLO-Y4 OR SCD-O OR HOCY AND native matrix; osteocyte OR osteocyte cell line AND mechanical loading; osteocyte AND low oxygen; osteocyte AND 3D. The systematic search was restricted to articles published in English before December 2018. The authors CZ and NB performed the literature search.

### Study Selection and Data Collection Process

All abstracts of the articles selected were read, and articles of potential interest were reviewed in detail (full text) by author CZ to decide on inclusion or exclusion from this review (see above). In case of disagreement, authors CZ and NB reviewed and discussed the article and a final decision on inclusion or exclusion was made through consensus.

### Data Extraction

Author CZ extracted information regarding osteocyte culture models from all included studies using a pre-determined template comprising of cell type, cell isolation protocol, animal model used, cell model, comparison, intervention techniques, outcomes. Given the heterogeneity of the characteristics studied in the included papers, a meta-analysis was not performed.

## Results

### Study Selection

**Fig. 1:**
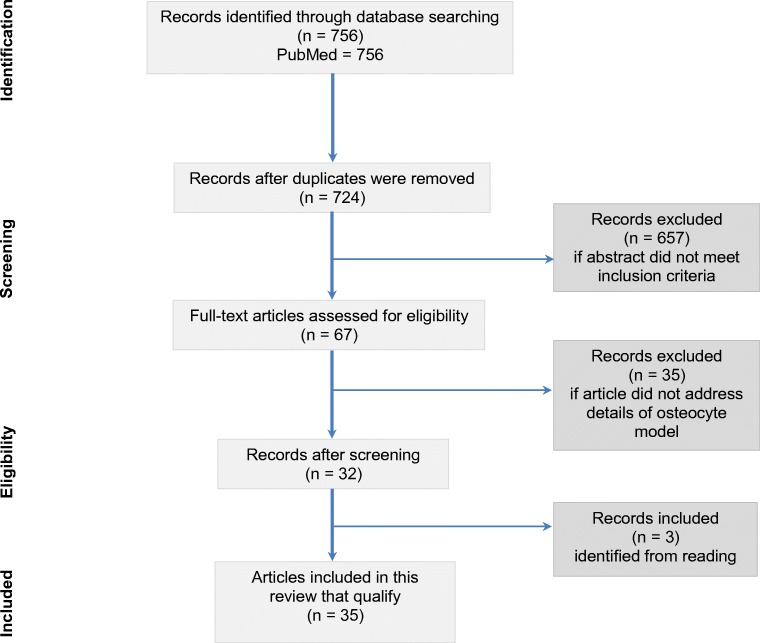
Study flow of the selection process of the articles used in this review

The literature search elicited a total of 756 articles. Thirty-two of these articles were duplicates and 657 articles were excluded since they did either not fit the aim of this review or they did not describe the culture method (Fig. 1). Sixty-seven articles were reviewed in full text, of which 32 articles met the inclusion criteria for the current review. After reading these articles, another three articles were found to be eligible for inclusion, resulting in a total number of 35 articles included in this study describing 3D-culture models (Table 1) [15••, 24••, 25••].

**Table 1:** Key reference articles of this review

Ref no.	Cell type	Species	3D model	Intervention	Outcome parameters	Significance
[24••]	MLO-Y4	Mouse	Collagen gel embedded osteocyte with titanium plate	Cyclic stress	Cell morphology, Cx43 expression, viability, apoptosis, RANKL expression, effect of CM on BMC	First study to apply mechanical loading on 3D cultured MLO-Y4
[25••]	PHOC	Human	BCP μ-beads-guided	Oxygen level	Cell morphology, spatial distribution, viability, mineralization, osteocyte/osteoblast marker expression	In vitro model with low oxygen level and 3D structure for osteocytes, mimicking precisely in vivo environment
[15••]	PHOC, MLO-A5	Human (PHOC), mouse (MLO-A5)	BCP μ-beads-guided	Cyclic compressive loading, PTH treatment	Gene and protein expression of osteocytes, Ca^2+^ oscillation	1st model replicating in vivo human primary osteocyte response to mechanical loading. Useful model to evaluate therapeutic agents for bone diseases

*CM* culture medium, *BMC* bone marrow cells, *PHOC* primary human osteocytic cells, *BCP* biphasic calcium phosphate, *μ-beads* microbeads, *PTH* parathyroid hormone

### Cell Lines

Since osteocytes are difficult to isolate from the mineralized bone, many investigators developed cell lines to simulate osteocyte-like cell behavior to study the role of osteocytes in mechanosensation and menchanotransduction. Currently, the most frequently utilized cell line is the murine long bone osteocyte Y4 (MLO-Y4), representing mature osteocytes [26]. Cell lines representing other developmental stages of osteocytes have been established to study osteocyte function and transition from osteoblast to osteocyte, i.e., the post-osteoblast, pre-osteocyte, MLO-A5 cell line [27], the post-osteoblast-to-late osteocyte, IDG-SW3 cell line [28], and the mature osteocyte, Ocy454 cell line [29]. These cell lines have their limitations, since they are modified to dedifferentiated stages enabling proliferation, while osteocytes in vivo do not proliferate.

MLO-Y4 has been established as an osteocyte-like cell line suitable to study the effects of mechanical stress on osteocytes and their relation with other bone cells. MLO-Y4 cells are developed from sequentially digested long bone cells derived from transgenic mice that show expression of SV40 large T-antigen oncogene driven by the osteocalcin promoter, which predominantly targets osteocytes [26]. MLO-Y4 cells are characterized by high proliferative capacity and maintenance of a dendritic morphology when kept in medium supplemented with 5% fetal bovine serum (FBS) and 5% calf serum (CS), which probably inhibits osteoblast growth [26]. Interestingly, MLO-Y4 cells exhibit a decreased growth rate on collagen-coated culture plastic surface compared to uncoated surface, regardless of serum components, which might be caused by the more dendritic morphology on collagen-coated surface [26].

MLO-Y4 cells and osteoblasts share the expression of osteopontin, osteocalcin, connexin 43 (Cx43), and CD44 [30–35]. Some molecules are hardly expressed by MLO-Y4 cells while they are produced by osteoblast-like cells such as alkaline phosphatase (ALP), osteoblast-specific factor 2, and type I collagen [36, 37]. Moreover, MLO-Y4 cells do not express the osteocyte-specific markers SOST and FGF23. However, they do respond to exogenous sclerostin treatment similarly as primary human osteocytes and in vivo osteocytes [28, 38, 39]. On the other hand, MLO-Y4 are able to express sclerostin induced by advanced oxidation protein products, thiazolidinediones, and TNF-related weak inducer of apoptosis [40–42]. This controversy might be caused by the different differentiation stages of MLO-Y4 cells.

To investigate osteocyte mechanosensitivity in osteocyte-like cells, the application of mechanical stimuli can be performed by pumping fluid over the cells thereby inducing fluid shear stress, and mimicking the interstitial fluid flow in the lacuno-canalicular network during loading [43]. The effect of frequency, peak shear stress amplitude, and total flow duration of fluid shear stress on osteocytes has been studied using MLO-Y4 cells to establish how osteocytes respond to different mechanical loading regimes [44, 45]. MLO-Y4 responds to fluid shear stress with higher amplitudes and frequencies with upregulation of COX-2 and downregulation of RANKL/OPG [45]. Besides fluid shear stress, a stokesian fluid stimulus probe also produces hydrodynamic forces in a singular cell [46]. In addition, optical tweezers and microneedle have been used to apply mechanical loading on a single cell [47]. Taken together, MLO-Y4 cells are useful to investigate osteocyte mechanoresponsiveness, but these cells exhibit some differences with osteocytes in vivo and some similarities with osteoblasts. Besides mechanical loading, the oxygen level is also very important for osteocytes activity. Osteocytes upregulate HIF-1α expression under hypoxia (2% O_2_) [48].

MLO-Y4 cells in 2D monolayer models do not fully mimic osteocyte function in vivo, since in vivo osteocytes are embedded in a calcified matrix, which is a 3D structure. Recently, several materials and techniques have been developed to mimic the environment of osteocytes in vivo in 3D models. One such model comprises MLO-Y4 cells suspended in a gel that is mixed with neutralized collagen solution and Matrigel™ [24••, 49]. To mimic the physiological loading sensed by osteocytes around an implant, a titanium plate embedded in the gel was subjected to cyclic stress for 24 h. In this model, MLO-Y4 cells connected by their processes in the collagen/Matrigel mixture like in the lacuno-canalicular network in vivo display increased Cx43 expression and cell apoptosis after cyclic stress treatment, similar to results observed in a mouse model [24••].

Other cell lines, MLO-A5, MLO-C2, and MLO-D1, have been developed from the same osteocalcin promoter-driven T-antigen transgenic mice as MLO-Y4, to examine the initiation of bone matrix mineralization [27]. These cell lines differ from MLO-Y4 in their differentiation stage as they are in a post-osteoblast, pre-osteocyte stage. MLO-A5 could be useful for bone mineralization and mechanical loading studies, since it is the only cell line that produces bone-like mineral in the absence of mineralization medium. Young primary osteocytes are more sensitive to stretching stimuli than mature osteocytes and osteoblast [50]. MLO-A5 has been utilized to explore an ex vivo 3D-model’s ability to mimic the environment and construction of osteocytes in their native matrix [51]. A 3D model of MLO-A5 mixed with biophasic calcium phosphate (BCP) microbeads coated with collagen type I, in a microfluidic device with osteogenic medium has been established, showing that MLO-A5 shows a significantly higher expression of mature osteocyte genes (SOST and FGF23) than in 2D-monolayer. MLO-Y4 cannot express these genes in 2D-monolayer, implying that the geometrical feature of osteocytes might affect their mechanosensation and mechanotransduction ability [28, 51].

IDG-SW3 is another cell line reported to fully express an osteogenic differentiation profile from late osteoblast to late osteocyte [28]. It expresses Dmp1-GFP shows mineralized extracellular matrix formation and produces E11/gp3 which is the earliest protein during differentiation from osteoblasts to osteocytes. IDG-SW3 expresses detectable SOST mRNA and sclerostin, which is inhibited by PTH. FGF23 is detected in IDG-SW3 and is increased by 1,25-dihydroxyvitamin D_3_. IDG-SW3 cultured with osteogenic differentiation medium is utilized as a model to study the role of HIF activation in oxygen sensor prolyl hydroxylase (PHD)-2 induced bone formation [52]. Whether IDG-SW3 cells are suitable for mechanosensitivity experiments remains unanswered.

The Ocy454 cell line replicates in vivo mature osteocytic functions [29]. Ocy454 cells express high levels of sclerostin in vitro in a short experimental time frame (i.e. 1 week) without adding differentiation factors [29]. Furthermore, Ocy454 in 3D respond to FSS and in 3D Alvetex™ polystyrene scaffolds to FSS and microgravity [29].

### Primary Osteocytes

**Fig. 2:**
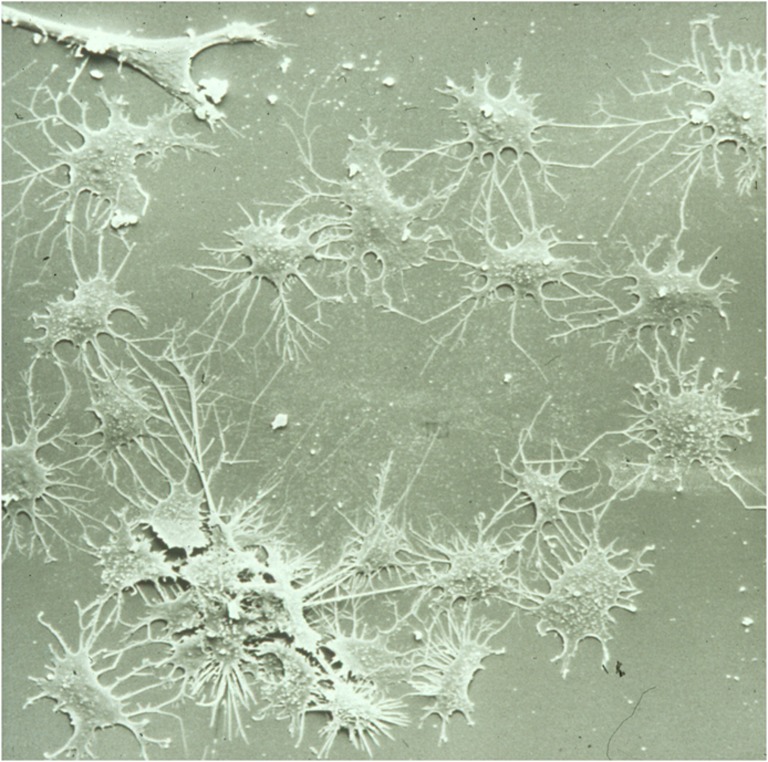
Scanning electron microscopy image of osteocytes, isolated from chicken bone, showing the typical characteristics of the cell bodies and their dendritic processes spread out over the surface contacting each other, thereby mimicking the osteocyte network in vivo

Primary osteocytes isolated from bone tissue are widely utilized to retain a complete osteocytic phenotype. Their activities are thought to more closely mimic osteocyte activities in vivo. The first available primary osteocyte was derived from chicken, which was used to study the role of osteocytes in mechanotransduction, and related signaling transduction pathways [5, 7, 53]. These osteocytes are purified from a heterogeneous bone cell population with the use of the osteocyte-specific monoclonal antibody (Mab) OB 7.3 bound to protein G-conjugated magnetic beads [53–55]. These osteocytes have a well-preserved osteocyte morphology with many stellate protrusions (Fig. 2). Compressive or tensile mechanical stimuli have been applied by indenting concave culture wells in a culture device [56]. The main disadvantage of the use of these primary chicken osteocytes was the limited availability of primers and antibodies for research.

The source of murine primary osteocytes is bone tissue, like calvaria or long bone [57, 58]. Most isolation protocols use several collagenase incubations with or without a decalcification incubation step [59–61]. Isolated cells of 7–9 fractions are considered primary osteocytes, as well as outgrowth from the bone tissue that remains after sequential digestion [59]. Cells derived from sequential digestion have a stellate shape with long processes. They express osteocalcin, SOST, and PHEX but lack or exhibit weak expression of ALP, suggesting that they are mature osteocytes. These cells have been demonstrated to be mechanosensitive since FSS-induced BMP-7 secretion prevented dexamethasone-induced apoptosis of osteocytes and altered NO production and Cox-2 mRNA expression [62, 63]. Besides FSS, flexible-bottom plate stretching is utilized to apply mechanical loading on primary osteocytes, which induces expression of c-fos and COX-2 in osteocytes [64]. Intermittent hydrostatic compression modulates ALP and type I collagen mRNA expression in neonatal mouse calvarial cells [65]. The currently most widely used method is the sequential digestion protocol by Stern et al. [59], which is derived from the combined methods of Gu et al. [66] and Van Der Plas et al. [53]. Cells obtained from early digestions express mostly osteoblast markers like ALP and COL1A1 but later digestions express more pre-osteocyte-specific marker E11/GP38 protein [59, 66]. The final digestions are osteocyte-enriched, and these cells express osteocyte-specific genes including SOST, COX-2, MEPE, PHEX, DMP1, but not FGF23 [59, 67]. However, FGF23 expression is observed in the cell outgrowth of cultured bone particles that are left-over after sequential digestion, but whether this is caused by the fact that FGF23 is a very late marker of mature osteocytes or by temporarily loss of FGF23 expression due to the collagenase and EDTA treatment remains unrevealed [59]. A limitation of the use of sequential digestions to obtain primary osteocytes is that it may lead to contamination of osteoblasts in cultures of longer duration.

Primary osteocytes can also be obtained by protocols culturing human bone-derived cells up to 5 weeks in mineralization medium to generate an osteocytic phenotype, called human primary osteocyte-like cells (hOCy) [39, 68]. Addition of strontium ranelate to these cultures induces the expression of osteocalcin, sclerostin, and DMP-1, thereby causing osteoblast-to-osteocyte transition with cells exhibiting an osteocyte-like morphology [68, 69]. These cells respond similar to sclerostin treatment as MLO-Y4 cells and osteocytes from SOST-transgenic mice in vivo [39].

Important limitations of 2D monolayer cell cultures are the lack of 3D cell distribution in the matrix as well as exposure to oxygen levels exceeding physiological levels in vivo. Moreover, mechanoresponsiveness can only be studied by applying fluid shear stress or cell stretching. A better model without these limitations is required to simulate the responsiveness of osteocytes to mechanical loading in vivo, which would be useful for better understanding the physiology of training patterns of athletes in vivo. 3D cell culture models could show a more physiologic mechano-response to loading. Recently, 3D models of primary osteocytes have been established [70]. In this 3D model, the osteocytes are seeded on biphasic calcium phosphate (BCP) microbeads with a diameter of 20–25 μm, enabling osteocytes to connect to each other with their dendritic processes, and thus simulating a 3D network [71]. Primary murine osteocytes cultured in this 3D model express late osteocyte markers such as SOST and FGF23 and show a non-proliferative behavior, like mature osteocytes [70]. A limitation of this model is that the murine osteocytes are damaged easily. Human primary osteocytes cultured in this 3D model show decreased ALP and increased FGF23 and SOST expression, while they barely proliferate [67]. Using in vitro BCP-guided 3D model, the mechano-response of osteocytes in vivo has been replicated for the first time [51]. Human primary osteocytes in this 3D model downregulate the mechanical loading-induced SOST expression, which is consistent with an in vivo study in rodents showing that mechanical stimulation of bone reduces osteocyte expression of sclerostin and another in vitro study showing Ocy454 in 3D Alvetex™ scaffolds respond to short-term mechanical overloading (i.e., 2 h) by reducing sclerostin expression [15••, 29]. To even better simulate the natural environment of the osteocytes, a hypoxia condition has been applied [25••]. One percent oxygen almost completely inhibits cell proliferation resulting in a more physiologic morphology and distribution of osteocytes and a synergistic upregulation of SOST and FGF23 expression [25••]. Interestingly, the thin surface cell layer of this hypoxic 3D tissue shows high ALP expression, while the cells in the interior express SOST and HIF1α, but not ALP. In 3D cultures under normoxia, cells are more proliferative and exhibit ALP expression from the surface to the interior, while they show no HIF1α and almost no sclerostin expression [25••]. This suggests that the 3D hypoxia model mimics the osteoblastic layer in native bone called endosteum. Hypoxia has been shown to facilitate the maintenance of an osteocyte phenotype in 2D monolayer culture with a distinct morphology. Hypoxia enhances mineralization and sclerostin expression but decreases ALP activity compared with normoxia [25••].

The most ideal model to mimic osteocytes in vivo maintains the osteocytes in their native matrix. However, this ideal model has not been extensively studied. Previous studies show that human bone chips in vitro release sclerostin and FGF23 during a 3-day culture period, with declining release over time [72]. Around 60% of the osteocytes, embedded in human trabecular bone in vitro, are alive after 7 days of culture and express SOST and FGF23 [73]. The viability of osteocytes in bone explants has been maintained for up to 4 weeks using a medium perfusion system [74]. Moreover, the amount of live osteocytes in a calf trabecular bone explant decreased within 8 days, and dynamic hydrostatic pressure enhances osteocyte viability [75]. These ex vivo studies provide a possible model to study osteocytes in their native matrix, and suggest that the surrounding calcified matrix could maintain the osteocyte physiological features and influence the mechanoresponsiveness of the cells. Mechanical stimulation in these models can be applied in many types of loading apparatuses like bioreactor systems [64, 75–82]. Living bone samples have been cultured and loaded for a long-term study (i.e., 22 days), simulating the in vivo situation [75].

## Discussion

In this review, several in vitro models of osteocytes are discussed, ranging from osteocyte cell lines and primary osteocytes, isolated and cultured in 2D monolayer, or in 3D bone-mimetic matrices or native matrix models. The different models are utilized to study different aspects of osteocyte function and behavior. This review aims to provide an overview of the different culture models to study osteocytes, by focusing particularly on investigations of the mechanosensitivity and mechanoresponsiveness of osteocytes.

Ideally, the investigated osteocytes resemble the natural situation in vivo as much as possible. The culture of osteocytes in their native matrix approximates the in vivo situation the most, since the osteocyte morphology, characteristic, and network are maintained. Furthermore, osteocytes in their native matrix enable to study how bone disease may affect osteocyte activity. Unfortunately, the number of cells is relatively low in this culture model and cannot be enhanced by stimulation of proliferation, thereby limiting the use of RNA isolation and RT-PCR techniques for gene expression studies. Moreover, the lifespan of osteocytes in a cultured bone chip is not completely known. After a 7-day culture period, 60% of the osteocytes in human bone chips are still alive [76]. Longer culture periods might be possible with maintenance of viable osteocytes, but this is currently unknown. Another issue while culturing osteocytes in their native matrix is the rather limited diffusion of chemicals into the bone tissue. This is especially difficult with larger proteins such as hormones or growth factors, and possibly also for pharma-therapeutics to be developed in the future. The investigation of the mechano-response in osteocytes in their native environment is also challenging. To date, there are no reports on the effects of mechanical loading on osteocytes cultured in their native matrix. Mechanical load might be applied during culture by compression or three-point bending in a bioreactor, but the use of special equipment is necessary.

3D models created by BCP microbeads in combination with low oxygen tension mimic the distribution and networking of osteocytes in vivo in order to acquire a non-proliferative state and expression of late osteocyte markers [67]. These features make this model very useful for relatively long-term studies on osteocyte communication with other bone cells as well as for studies on the response to mechanical loading. Since the structure of this model is not a natural calcified matrix, it may be suitable for investigations on the distribution of hormones, signaling molecules, and pharmaco-therapeutics. A shortcoming of 3D models containing BCP is that the osteocytes are embedded in a 3D construction, making the microscopic observation of the cells difficult.

2D monolayer osteocyte cultures are not completely resembling the natural osteocyte environment, but they are quite convenient to use regarding the culture condition requirements, the cell growth potential, flexible intervention, and possibility of close microscopic observation. In addition, the cell morphology can be recorded using live microscopy during specific interventions. Obviously, 2D monolayer culture has the disadvantage that osteocytes dedifferentiate towards osteoblasts, and primary cell isolation procedures may result in a mixture of osteoblasts and osteocytes in different differentiation stages, which might affect osteocyte behavior [25••].

The behavior of osteocytes might be source-dependent. Since cell lines are gene-transfected to become immortalized cells, the gene expression profiles of the cells as well as the cellular physiology is changed [22, 23], possibly leading to an inconsistency with primary osteocyte mechanoresponsiveness. Different species also have an influence on osteocyte behavior, since osteocytes from mice cannot reliably recapitulate human osteocytes [25••]. This may result in limited extrapolative value of mechano-response studies for human bone disease and therapies. Note that human primary bone cells are easier to digest than murine primary bone cells and can be stored up to four passages in liquid nitrogen [67]. This is an enormous advantage, since the cells isolated from human bone tissue after surgery can now be easily preserved for future in vitro experiments. The isolation procedure of osteocytes from their bone matrix may affect the purity of the osteocyte culture. Three different isolation methods have been described, i.e., antibody connected to magnetic beads, long-term culture in mineralization medium, and sequential digestion. The use of MAb OB 7.3 resulted in the highest purity of mature osteocytes, but this isolation procedure is limited to chicken bone because of the source of the antibody [53, 54]. Selective medium and sequential digestion also provide a highly pure osteocyte population, but different osteocyte differentiation stages are isolated during this procedure, which may influence the mechanosensitivity of the entire cell population [59, 67, 69].

In conclusion, several 2D and 3D osteocyte culture models are available for the investigation of osteocyte mechanosensitivity, which all have their advantages and disadvantages or limitations. The choice of the model to be used should be determined by the study objective. Although it is clear that osteocytes cultured in their native matrix resemble osteocytes in their in vivo situation most closely, there are also advantages to use primary osteocytes or osteocyte cell lines either in 3D models with BCP or in 2D monolayer models.
